# Mitochondrial Dysfunction in Systemic Lupus Erythematosus: Insights and Therapeutic Potential

**DOI:** 10.3390/diseases12090226

**Published:** 2024-09-23

**Authors:** Anastasia V. Poznyak, Nikolay A. Orekhov, Alexey V. Churov, Irina A. Starodubtseva, Dmitry F. Beloyartsev, Tatiana I. Kovyanova, Vasily N. Sukhorukov, Alexander N. Orekhov

**Affiliations:** 1 Institute for Atherosclerosis Research, Osennyaya 4-1-207, 121609 Moscow, Russia; kovyanovat@gmail.com; 2 Laboratory of Angiopathology, Institute of General Pathology and Pathophysiology, 8 Baltiiskaya Street, 125315 Moscow, Russia; www.fuper@gmail.com (N.A.O.); achurou@yandex.ru (A.V.C.); vnsukhorukov@gmail.com (V.N.S.); 3 Russian Gerontology Clinical Research Centre, Institute on Aging Research, Pirogov Russian National Research Medical University, Russian Federation, 16 1st Leonova Street, 129226 Moscow, Russia; 4 Department of Polyclinic Therapy, N.N. Burdenko Voronezh State Medical University, 10 Studencheskaya Street, 394036 Voronezh, Russia; starodubtsevairina1@gmail.com; 5 Vascular Surgery Department, A.V. Vishnevsky National Medical Research Center of Surgery, 27 Bolshaya Serpukhovskaya Street, 117997 Moscow, Russia; beloyar@rambler.ru

**Keywords:** autoimmune disease, inflammation, mitochondria, rheumatic diseases, immune responses

## Abstract

Systemic lupus erythematosus (SLE) is a complex autoimmune disorder characterized by the presence of various serum autoantibodies and multi-system effects, predominantly affecting young female patients. The pathogenesis of SLE involves a combination of genetic factors, environmental triggers, and pathogen invasions that disrupt immune cell activation, leading to the release of autoantibodies and chronic inflammation. Mitochondria, as the primary cellular powerhouses, play a crucial role in SLE development through their control of energy generation, reactive oxygen species (ROS) production, and cellular apoptotic pathways. Dysregulation of mitochondrial structure and function can contribute to the immune dysregulation, oxidative stress, and inflammation seen in SLE. Recent research has highlighted the impact of mitochondrial dysfunction on various immune cells involved in SLE pathogenesis, such as T-lymphocytes, B-lymphocytes, neutrophils, and plasmacytoid dendritic cells. Mitochondrial dysfunction in these immune cells leads to increased ROS production, disrupted mitophagy, and alterations in energy metabolism, contributing to immune dysregulation and inflammation. Moreover, genetic variations in mitochondrial DNA (mtDNA) and abnormalities in mitochondrial dynamics have been linked to the pathogenesis of SLE, exacerbating oxidative stress and immune abnormalities. Targeting mitochondrial function has emerged as a promising therapeutic approach for SLE. Drugs such as sirolimus, N-acetylcysteine, coenzyme Q10, and metformin have shown potential in restoring mitochondrial homeostasis, reducing oxidative stress, and modulating immune responses in SLE. These agents have demonstrated efficacy in preclinical models and clinical studies by improving disease activity, reducing autoantibody titers, and ameliorating organ damage in SLE patients. In conclusion, this review underscores the critical role of mitochondria in the pathogenesis of SLE and the potential of targeting mitochondrial dysfunction as a novel therapeutic strategy for improving outcomes in SLE patients. Further investigation into the mechanisms underlying mitochondrial involvement in SLE and the development of targeted mitochondrial therapies hold promise for advancing SLE treatment and enhancing patient care.

## 1. Introduction

SLE is a serious autoimmune disorder which is mostly characterized by numerous serum auto-antibodies (AAbs) and multi-system effects. This disorder is found mainly in young female patients. Epidemiologic studies show that systemic lupus erythematosus affects an average of 5.14 people in 100,000, with an annual prevalence ranging from 0 to 241 worldwide. Although, differences in environment, study design, and populations lead to different results in the studies. The precise pathogenesis of systemic lupus erythematosus is still not fully understood [1,2]. Although, it seems that this disorder is related to genomic characteristics, environmental conditions, and pathogen invasions. Such triggers may stimulate abnormal activation of ICs (immune cells), which reduces immunity tolerance towards autoantigens, facilitates the release of AAbs, and disrupts IC deposition and clearing. Consequently, this can lead to constant inflammation and CNS, hematological and renal disorders [3].

In the past years, significant achievements were made in diagnostics and therapy of systemic lupus erythematosus. It is noteworthy that generative research targeting particular pathways and discoveries of novel agents considerably contributed to the systemic lupus erythematosus therapy advances. Although, a remission is still not reached and individuals suffering from this disorder face dire prognoses. One of the reasons for it might be the complex clinical manifestations of systemic lupus erythematosus, its variety, and the difficulty in predicting organ damage [4,5]. Systemic lupus erythematosus therapy involves steroids, immune suppressants, and malaria medications. Although, prolonged application of those drugs may lead to non-convertible organ failure and provoke some serious negative effects, e.g., myelosuppression and infection. Hereby, a close investigation of cell and molecule immune aberrances in systemic lupus erythematosus development to find novel targets and therapeutic approaches is required with expedition [6].

Mitochondria (Mts) are the main cellular powerhouses; they have two membranes—outer and inner membranes (MOM and MIM). Mt generates adenosine triphosphate via OXPHOS, thus stimulating cell function and defining its fate, i.e., differentiation of cells, activation, and apoptosis. OXPHOS is essential for adenosine triphosphate production since it is underpinned by 5 enzyme complexes on ETC. A proton gradient generated by enzyme complexes 1–4 is used by the fifth complex for ADP phosphorylation to produce adenosine triphosphate. Simultaneously, upon the oxidative phosphorylation, Mts generate ROS [7,8]. In a cell, Mts are the main source or reactive oxygen species. Reactive oxygen species take part in reduction–oxidation balance and regulate cellular apoptotic processes, thus inducing development of systemic lupus erythematosus and consequent organ failure. Overproduction of reactive oxygen species can damage cell structure, disrupt its activity, and trigger activation of numerous pathways, thus enhancing inflammation in systemic lupus erythematosus [9,10].

In addition, Mts are critical in cellular iron metabolism and Ca^2+^ regulation. These organelles are the major players in cell physiology and metabolism. Emerging evidence indicate that aberrant Mt structure and activity may provoke or aggravate development of systemic lupus erythematosus via multiple mechanisms, such as modification of IC metabolic processes and structural disorders of an organelle, which can elevate the production of proinflammatory mediators and amplify activation of ICs [11].

Our review emphasizes the important role of Mt structure and function in systemic lupus erythematosus development, e.g., mitoDNA alterations, Mt autophagy, genome polymorphisms, Mt dynamics, changes in biogenesis and energetic metabolic process, OS, and various cellular death pathways. We sum up the recent studies results on the pathophysiological role of Mts which investigate their impact on the systemic lupus erythematosus development and possibilities of targeted treatment approaches. These findings offer a new knowledge on the etiology of this disorder and enable further advancements.

## 2. Anatomy, Role and Activity of Mitochondria

Mitochondria are essential cellular organelles known as the “powerhouses” of the cell, primarily responsible for generating adenosine triphosphate (ATP) through oxidative phosphorylation (OXPHOS) [12]. They have a unique double-membrane structure consisting of an outer membrane and a deeply folded inner membrane (the cristae), which increases the surface area for ATP production [13]. Mitochondria also contain their own mitochondrial DNA (mtDNA), which is highly sensitive to oxidative stress [14].

In addition to energy production, mitochondria play a critical role in regulating cellular metabolism, calcium homeostasis, and apoptosis [15]. They control the production of reactive oxygen species (ROS), which are vital for signaling but can cause cellular injury in excess [16]. Mitochondrial dysfunction, characterized by impaired energy metabolism and increased ROS production, has been implicated in the pathogenesis of autoimmune diseases like systemic lupus erythematosus (SLE) [17].

Emerging evidence suggests that mitochondrial abnormalities contribute to immune dysregulation observed in SLE [18]. Dysfunctional mitochondria can disrupt mitophagy—the process by which damaged mitochondria are removed—leading to increased inflammation and autoantibody production [19]. For example, in SLE, altered mitochondrial dynamics and ROS overproduction in immune cells, such as T-lymphocytes and B-lymphocytes, exacerbate autoimmune responses [20].

Thus, understanding the role of mitochondria in energy metabolism and immune function is crucial for elucidating their involvement in SLE and identifying potential therapeutic targets [21,22,23,24,25,26].

## 3. Mitochondrial Function Impairment in Systemic Lupus Erythematosus

### 3.1. Reduction–Oxidation Alterations: A Vicious Cycle

Normally, Mts produce a modest amount of mitochondrial reactive oxygen species to mediate different signaling pathways and cell metabolic processes. Although, elevated concentrations of mitochondrial reactive oxygen species may induce pathological processes and impair Mt function, which leads to cell injury [26,27,28,29]. GSH peroxidase is an enzyme that provides a balance of GSH and GSH disulfide. GSH peroxidase detoxicates fat peroxides and removes reactive oxygen species from Mt. In case of OS, Mt DNA may be released from Mt to cytosol [30,31]. 8OHdG is believed to indicate DNA injury caused by oxidants. Resent research demonstrated that 8OHdG is significantly abolished by reducing Mt DNA copies in subjects with systemic lupus erythematosus. In parallel, oxidized Mt DNA, which can provoke a major immune response, enhances production of mitochondrial reactive oxygen species and facilitates inflammation. Moreover, impaired Mt anti-oxidant enzymes in ICs of subjects with systemic lupus erythematosus are able to exacerbate OS in a vicious circle. Hereby, mitophagy modulated by OS was proven to be essential in genomic propensity and activation of ICs in systemic lupus erythematosus [32,33]. In Table 1, we summarized the role of mitochondrial impairments in SLE.

### 3.2. Genetic Mechanism of Mitochondrial Function Impairment

Mt DNA is placed very close to the inner membrane and cristae, and there are no histones and introns, which makes it very sensitive to internal damage, mainly because of the ROS produced by Mt complexes upon oxidative phosphorylation [14,15]. Considering all that was stated earlier and because of lower efficacy of the DNA repair mechanism, mutations of Mt DNA occur ten to twenty times more frequently than mutations of nDNA. Changes in its structure and sequence occur very often. MtDNA–CN ranges between five and ten. Numerous Mt DNA copies in a cell and its exceptional sensitivity to changes facilitates the coincidence of different Mt DNA populations in one cell (mutated Mt DNA and wild-type Mt DNA) [16,17]. This paradox is called heteroplasmy, in contrast to homoplasmy—occurrence of only one type Mt DNA in one cell. The heteroplasmy rate is calculated as the ratio of mutated Mt DNA to total Mt DNA [18,19].

Genetic propensity and numerous other factors are involved in the systemic lupus erythematosus development. Many GWAS demonstrated that some Mt SMPs account for the increase in vulnerability to systemic lupus erythematosus. For instance, adenosine triphosphate synthase 5/6, D310, D-loop, ND1, and ND-2 were shown to take part in Mt genesis and mitophagy, which indicates that Mt DNA polymorphisms increase vulnerability to systemic lupus erythematosus. Pathogenetic AAbs and ICs buildup in tissues lead to multiorgan failure in systemic lupus erythematosus. AAbs against Mt contents show that Mt are major anti-genic inducers which trigger immune response in systemic lupus erythematosus [34,35]. Different AAbs, e.g., anti-Mt, anti-whole Mt, anti Mt DNA, anti-MtRNA, and ACA, were produced by targeting different Mt contents, such as Mt surface, DNA, RNA, and MIM. This evidence implies that Mt are an essential source of auto-antigens which facilitate development of systemic lupus erythematosus. Moreover, available data indicates that Mt function impairment and aberrant mitophagy lead to reduction–oxidation damage of Mt DNA, excessive genesis of mitochondrial reactive oxygen species, disturbed OS balance, and inflammation pathways activation. The above-mentioned factors contribute to manifestation and progression of systemic lupus erythematosus [36,37].

### 3.3. Mitochondrial Function Impairment in ICs

**Figure 1:**
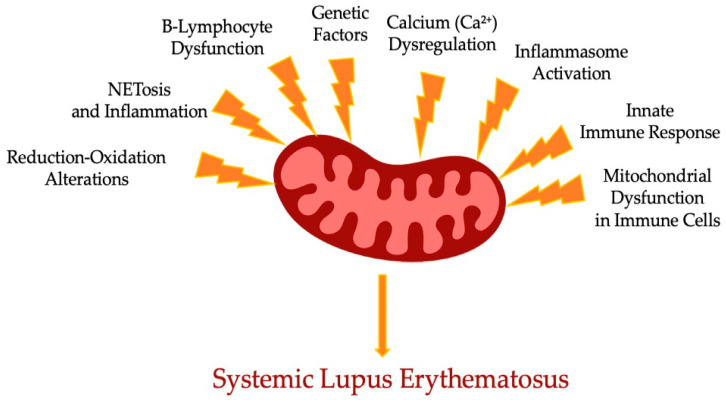
Scheme of complex role of mitochondria dysfunction components in the SLE.

It is believed that Mt appeared as a result of bacterial endosymbiosis. Moreover, modulation of intrinsic energy in Mt is crucial for Mt metabolic processes and the signaling cascade of immunity response. Reduction–oxidation damage caused by Mt function impairment promotes activation of different inflammation pathways in innate and acquired immunity. Research has been conducted on several cell-signaling pathways which control mitophagy in systemic lupus erythematosus, such as mTOR Complex 1, 5′ AMP-activated protein kinase, cryopyrin, inflammasome, and cGAS–TMEM173 pathways [38,39]. In Figure 1, we schematically summarized the range of mechanisms affecting the mitochondrial dysfunction leading to SLE.

## 4. Mitochondrial Function Impairment in Acquired Immunity

Mt function impairment was found in SLE T-lymphocytes and is characterized by mitochondrial reactive oxygen species overproduction, increased membrane potential, and reduced mitophagy and GSH level. Furthermore, OX attenuates proximal signaling of TCR, activation of pathways, and exhaustion-related expression of genes program. In addition, prolonged enhancement of antigens leads to a non-convertible T-lymphocyte exhaustion and disrupts mitophagy, leading to excessive genesis of mitochondrial reactive oxygen species and expression of auto-antigens [40,41].

A number of signaling pathways facilitate the scavenging of injured Mt through mitophagy, inhibiting mitochondrial reactive oxygen species aggregation. M-TOR pathway activation, which relies on reduction–oxidation, is important for modifying T cell receptor signal transmission and T-lymphocyte differentiation in subjects with systemic lupus erythematosus. Moreover, T-lymphocyte activation seems to need Mt to be located in immunological synapse. Although, m-TOR activation reduces the level of T-lymphocyte surface receptor/CD3 zeta chain while increasing levels of protein TK and FC gamma chain receptor [30,42]. Subsequently, this elevates the Ca^2+^ efflux in SLE T-lymphocytes via degrading by lysosomes, which relies on HRES1/Rab-4. The abnormal changes in Calcium can be the reason for inadequate activation in SLE T-lymphocytes. It is noteworthy that overexpression of small GTPase HRES1/Rab-4 drains the DRP-1, resulting in mitophagy downregulation in T-helper cells in SLE. On the contrary, Rab-4 suppressed with 3PEHPC repairs DRP-1 functioning through an m-TOR-independent pathway, revoking Mt aggregation in SLE T-lymphocytes [43].

Enhanced efflux of Ca^2+^ can also stimulate OS-dependent activation of T-lymphocytes. e.g., Ca^2+^-mediated phosphatase CaN induces de-phosphorylation of NFAT. Intranuclear nuclear factor of activated T-lymphocytes, together with such TF as activator protein 1, nuclear factor κB, and Oct1, stimulates IL2 expression. Notably, suppression of Rab-4A ameliorates mitophagy in T-lymphocytes, T-lymphocytes activation, and AAbs genesis in systemic lupus erythematosus. This indicates that repairment of Mt function, impaired by poor mitophagy and excessive expression of mitochondrial reactive oxygen species, returns the phenotypes of T-lymphocytes in systemic lupus erythematosus to normal [44,45]. Moreover, a number of studies showed that m-TORC-1 suppression by rapamycin (sirolimus) or additional intake of NAC (anti-oxidant and GSH precursor) demonstrate positive results and ameliorate inflammatory processes in systemic lupus erythematosus subjects. It is noteworthy that Mt aggregation drains memory T-lymphocytes and T-regs in lupus. In fact, reduced T-regs are unable to keep self-tolerance and immunoinhibiting activity in systemic lupus erythematosus, while additional intake of T-regs reduces inflammation and systemic lupus erythematosus progression in mouse models [46,47]. In the context of Mt energy metabolic processes, oxidative phosphorylation, through 5′ AMP-activated protein kinase activation, is more needed for these cells than T_h_17 cells. Metformin is able to normalize T regs immunity regulation by targeting 5′ AMP-activated protein kinase activation and inducing activation of signal transducer and activation of transcription 1, which relies on AMPK in systemic lupus erythematosus [48].

There is still not enough knowledge on the mitochondrial immune metabolic processes of B-lymphocytes in lupus. Nevertheless, the enhanced differentiation of CD-27^+^IgD^+^-unswitched memory B-lymphocytes into D-27^hi^CD-38^hi^ plasmablasts coincides with m-TORC-1 activation. The enhanced activation of m-TORC-1 directly correlates with excessive genesis of AAbs and cytokines in B-lymphocytes in lupus. Interestingly, inhibition of B-lymphocytes hyperreactivity was detected when using rapamycin in lupus [49]. Accordingly, m-TOR inhibition by the deletion of regulatory-associated m-TOR proteins, important signal adapters, suppresses cellular differentiation in plasma in mice with SLE. Earlier studies have demonstrated that LLPCs need slow production of adenosine triphosphate via 5′ AMP-activated protein kinase signaling and mitochondrial oxidative phosphorylation. Metformin was proven to improve systemic lupus erythematosus manifestations via blockade of B-lymphocyte differentiation into plasma cells and extension of germinal center by changing AMPK/m-TOR/STAT-3 signaling [50,51]. In Table 2, we summarized an impact of mitochondrial dysfunction in various cell types.

## 5. Mitochondrial Function Impairment in Innate ICs

Neutrophils and pDCs generate interferon alpha when nucleioacid–protein immune complexes are triggered. Interestingly, overproduction of interferon alpha, which is detected in >50% of subjects with systemic lupus erythematosus, promotes the expression of IFN-genic DNA, such as Mt DNA, from neutrophils, thus creating an auto-antigen/AB immune complex. Although, Mt degrading can be impaired when exposed to interferon alpha/ribonucleoprotein immune complex, which results in aggregation of excessive oxidized Mt DNA inside Mt and consequently outside of Mt, possibly triggering plasmacytoid dendritic cells [52,53,54]. Due to oxidized Mt DNA AAb presence, Mt DNA is a powerful antigen which promotes the genesis of AAbs in lupus. Moreover, the important biomarker of DNA oxidation, 8OHdG, is increased in the bloodstream of subjects with lupus. Mt DNA, which acts as a damage-associated molecular pattern, enhances the genesis of interferon alpha, leading to activation of the cytosolic deoxyribonucleic acid-sensing cGAS-TMEM173 and NLRP-3-signaling pathways. Impaired mitophagy and subsequent Mt-DNA-dependent DNA-sensing activation can possibly turn out to be a major step in systemic lupus erythematosus development [55].

NETs are structures containing DNA and chromatin designed to kill foreign microorganisms. The RNP immune complex and interferon alpha promote the activation and release of NETs via the activation of cGAS–TMEM173 pathway, Mt hyperpolarization, and excessive genesis of mitochondrial reactive oxygen species in lupus. It is noteworthy that Mt ROS-mediated and -oxidized Mt DNA trapped inside NETs, which are released onto the surface or neutrophils, provoke interferon genesis. Hereby, mitochondrial reactive oxygen species suppression decreases NETosis by suppressing Mt respiration in mouse model of SLE. These findings support the idea that Mt function and reactive oxygen species production contribute to the development of systemic lupus erythematosus [56].

Moreover, elevated concentrations of mitochondrial reactive oxygen species and enhanced OS were found in lupus monocytes, which exhibit interferon alpha signature. A defective degrading process of interferon-alpha-mediated Mt DNA results in elevated Mt membrane potential in lupus monocytes, STING-dependently stimulating their auto-reaction [57]. Furthermore, blood monocytes differentiation into auto-inflammatory DCs while interferon alpha is present stimulates activation and expanding of auto-reactive lymphocytes, partly modulating an acquired immunity. Interestingly, plasmacytoid dendritic cells are responsible for massive interferon alpha production in lupus, and interferon alpha release from dendritic cells mediated by Toll-like receptor 9 is decreased by the m-TOR suppressant that controls plasmacytoid dendritic cells activation and differentiation through modulation of Mt genesis and energy metabolic processes. Therefore, normalizing Mt DNA and mitophagy restoration by anti-oxidant therapy is a promising approach for systemic lupus erythematosus [58].

Cryopyrin and protein absent in melanoma 2 are DNA sensors which, together with ASC (apoptosis-related speck-like protein) and procaspase1, provoke the genesis of various downstream proinflammatory cytokines, such as interleukin 1 beta and interleukin 18, and stimulate pyroptosis. Numerous models of activation of cryopyrin inflammasome were detected in auto-immune disorders. However, the most prevalent model involves the activation of cryopyrin inflammasome with the reactive oxygen species being produced [59]. Scavenging of injured Mt by reducing OS and enhancing mitophagy is required for limited cryopyrin activation. Furthermore, mitochondrial reactive oxygen species and Mt DNA are essential for optimal activation of cryopyrin inflammasome and accompanying flux of Ca^2+^. Mt are critical for cellular accumulation of Ca^2+^ and are very important for modulation of Mt homeostasis. Notably, Ca^2+^ influx, in turn, induces mitochondrial reactive oxygen species and Mt DNA release to increase cryopyrin activation. MAP1LC3B/BECN1-modulated autophagy and NOD-2/RIPK-2-dependent mitophagy facilitate the removal of injured Mt and therefore downregulate activation of cryopyrin inflammasome [20,60].

Numerous trials demonstrated that neutrophil extracellular traps mediate cryopyrin inflammasome activation in macrophages of SLE subjects, thus stimulating genesis of interleukin 1 beta through reactive oxygen species and potassium efflux. Notably, inadequate scavenging of injured Mt leads to excessive release of Mt DNA, mitochondrial reactive oxygen species and CL, as well as excessive potassium efflux. This, in turn, activates cryopyrin inflammasome. Although, administering agents scavenging mitochondrial reactive oxygen species in mouse model of SLE considerably reduces inflammasome-associated expression of genes and expression of mature interleukin 18 [61,62]. And vice versa, trials have revealed that inflammasome alterations related to function impairment are in correlation with systemic lupus erythematosus progression. This could be due to the need for homeostatic inflammasome activity to balance protection and destruction caused by immune responses. Furthermore, defections in lysosome degradation of auto-phagosomes together with cargo are unable to remove Mt DNA and lysosomal Toll-like receptor 7 activation. It is noteworthy that a deficit of Mt-associated GTPases, such as IFI1, can result in maintained interferon activation, enhanced cGAS-TMEM173 activation, and suppressed autophagy in multiple autoimmune disorders [63,64].

## 6. Prospects of Mt-Targeted Treatment in Systemic Lupus Erythematosus Subjects

Therapy for systemic lupus erythematosus usually involves steroids, broad spectrum immunosuppressive medication, and malaria medication. Although, long-term application of such therapy may cause non-convertible organ failure, myelosuppression, and infections. A spike in targeted studies and the discovery of novel agents considerably improved therapy options for systemic lupus erythematosus. Interestingly, multiple trials emphasized the importance of Mt in lupus development. Hereby, Mt is considered a major therapeutic target for lupus. Subsequently, discovering new Mt-targeted ways of treatment, e.g., anti-oxidant therapy and metabolic therapy targeting lysosomal degradation, and investigating applications of existing agents are promising paths to improve systemic lupus erythematosus therapy [65]. In Table 3, we summarized the data on mitochondrial-targeted therapies for systemic lupus erythematosus.

### 6.1. Sirolimus (Rapamycin)

Sirolimus, originally identified as an antifungal agent derived from Streptomyces hygroscopicus, has gained recognition as a powerful inhibitor of the mechanistic target of rapamycin (mTOR) [66,67]. This unique mechanism functions primarily through the suppression of mTORC1, leading to a significant decrease in T-lymphocyte activity [68,69,70]. As a result, sirolimus has shown numerous beneficial effects in preclinical SLE models, including reductions in proteinuria, improvements in renal function, decreased levels of anti-dsDNA antibodies, and inhibition of antiphospholipid antibody (APLA) formation. Subsequent research on lupus patients has further highlighted sirolimus’s ability to enhance mitochondrial function and substantially lower lupus disease activity [71,72]. As a therapeutic strategy, sirolimus presents a promising avenue for managing systemic lupus erythematosus (SLE), especially in patients with renal complications where traditional immunosuppressive therapies may fall short [73,74]. Clinical trials have substantiated these findings, revealing significant reductions in proteinuria and anti-dsDNA antibody titers in patients treated with sirolimus, indicating its potential to not only improve kidney health but also to attenuate overall disease severity [75,76]. Ongoing studies are focused on refining treatment protocols by exploring optimal dosing regimens and combinations with other immunosuppressants to maximize efficacy. This accumulating evidence positions sirolimus as a critical component of future treatment paradigms aimed at achieving better outcomes for individuals suffering from SLE [77].

### 6.2. N-Acetylcysteine (NAC)

N-acetylcysteine (NAC) is recognized as a precursor to glutathione, an essential anti-oxidant that plays a pivotal role in the body’s defense against oxidative stress [78,79]. By facilitating the replenishment of intracellular glutathione levels, NAC not only enhances anti-oxidant capacity but also exhibits a unique ability to suppress T-lymphocyte activity through mTORC1 inhibition [80,81]. Notably, research by Doherty and colleagues highlighted NAC’s selective suppression of mitochondrial electron transport chain complex I, which helps to decrease oxidative stress specifically in individuals with lupus [82]. Studies in SLE murine models have demonstrated that NAC effectively lowers serum levels of anti-double-stranded DNA antibodies, mitigates symptoms associated with lupus nephritis, and prevents relapses of the disease [41,83]. Clinically, NAC has shown substantial efficacy in alleviating symptoms of SLE, making it a promising adjunctive therapy in the disease management landscape. Recent clinical trials have underscored its potential benefits, revealing significant reductions in disease activity and improvements in renal function, particularly for patients grappling with lupus nephritis [47]. These findings underscore NAC’s role not only in symptom management but also in addressing critical aspects of disease pathology. As research progresses, further investigations will seek to clarify NAC’s long-term efficacy and safety, while exploring its integration into combination therapies, ultimately enhancing treatment strategies for systemic lupus erythematosus [84,85].

### 6.3. Coenzyme Q_10_ (CoQ_10_)

Coenzyme Q10 (CoQ10) is a potent anti-oxidant that plays a crucial role in neutralizing free radicals in the cytoplasm, thereby shielding cell membranes and cellular components from oxidative stress [86]. Within mitochondria, CoQ10 is integral to electron transport, facilitating electron transfer from complexes I and II to complex III [86,87]. Research has highlighted the effectiveness of mitoquinone mesylate, a synthetic analog of CoQ10, in suppressing the production of mitochondrial reactive oxygen species. This action significantly diminishes the activation of neutrophil extracellular traps (NETs), reduces the generation of interferon alpha, and curtails kidney immune complex deposition, all of which contribute to the disease activity observed in MRL-lpr SLE mouse models [88,89]. Additionally, CoQ10 has shown promise in alleviating autoimmune inflammation in lupus patients. Studies utilizing idebenone, another synthetic analog of CoQ10, have reported beneficial effects in modulating immune responses, protecting against organ failure, and enhancing mitochondrial function [90,91]. These findings underscore CoQ10’s potential as a therapeutic strategy in managing systemic lupus erythematosus (SLE), particularly through its ability to combat oxidative stress. Preliminary clinical trials have suggested that CoQ10 may lead to significant reductions in reactive oxygen species production and improvements in immune function among SLE patients [92,93]. With encouraging outcomes from initial studies, ongoing and future research efforts are focused on further clarifying the therapeutic benefits of CoQ10 and establishing its role in a comprehensive treatment regimen for SLE, paving the way for enhanced patient care and management [94,95].

### 6.4. Metformin

Metformin, originally developed as a medication for type 2 diabetes, has recently gained attention for its potential role as an immunomodulator in patients with systemic lupus erythematosus (SLE) [79,96]. Emerging research indicates that metformin can normalize the redox balance in T-lymphocytes of lupus patients, enhancing the immune response regulation [97]. The drug also appears to inhibit the differentiation of B-lymphocytes into plasma cells and curtail the proliferation of germinal centers through modulation of the AMPK–mTOR–STAT-3 signaling pathway. In murine models of SLE, metformin has demonstrated significant efficacy in decreasing the production of reactive oxygen species, thereby reducing the leakage of mitochondrial DNA into neutrophil extracellular traps, which can exacerbate autoimmune inflammation [96,98]. This immunomodulatory effect has been associated with a reduction in disease flare-ups and a decrease in reliance on corticosteroids, underscoring metformin’s potential as a vital component of new therapeutic approaches for lupus management. Particularly beneficial for patients with concurrent metabolic syndrome, metformin’s ability to improve insulin sensitivity may address both metabolic and autoimmune dimensions of the disease, contributing to enhanced overall health outcomes [99,100]. Ongoing clinical trials are exploring metformin’s effectiveness in lowering flare rates and improving the quality of life for SLE patients, with early results showing promise in reducing disease activity indices. As research continues to unfold, metformin could play a crucial role in the evolving landscape of SLE treatment strategies, offering new hope for better management of this complex autoimmune condition [101].

### 6.5. Hydroxychloroquine (HCQ)

Hydroxychloroquine (HCQ) was initially introduced as a treatment for malaria but has since emerged as a cornerstone therapy for systemic lupus erythematosus (SLE), showcasing significant efficacy in this complex autoimmune condition. While the full spectrum of its effects remains to be fully elucidated, emerging evidence suggests that HCQ can modulate the mitochondrial anti-oxidant system that is activated by T-cell receptor cross-linking [102,103]. This modulation may lead to increased concentrations of reactive oxygen species in the mitochondria, thereby limiting the proliferation of T-helper cells through augmented oxidative stress responses. Additionally, HCQ functions as a powerful immunomodulator by suppressing the activation of Toll-like receptors 7 and 9 in antigen-presenting cells (APCs) [104,105]. This inhibition leads to a decrease in the production of proinflammatory cytokines, potentially slowing the binding of cyclic GMP–AMP synthase to cytosolic DNA and further diminishing the expression of inflammatory mediators. Clinically, HCQ is highly valued for its anti-inflammatory properties, helping to reduce disease activity, improve patient symptoms, and provide protective effects against organ damage, significantly enhancing long-term patient outcomes [106]. Numerous clinical studies have reinforced HCQ’s benefits in managing SLE, contributing to improved quality of life and survival rates for patients. Ongoing trials seek to better define its mechanisms of action and optimize dosing strategies, with the goal of refining treatment protocols and further improving the overall management of SLE. As research continues to expand our understanding of HCQ’s role, it remains a crucial element in the therapeutic landscape for lupus [107,108].

### 6.6. 3PEHPC

3PEHPC serves as a suppressant of Rab protein geranylgeranyltransferase, revealing a fascinating therapeutic potential, particularly in the context of systemic lupus erythematosus (SLE). When administered to SLE-vulnerable mice, 3PEHPC demonstrated noteworthy effects, including the restoration of dynamin-related protein 1 (DRP-1) generation, decreased mitochondrial deposition in T-lymphocytes, and a reduction in the production of antinuclear antibodies [109,110]. Additionally, it led to lower levels of proteinuria, alleviating symptoms associated with nephritis and contributing to improved kidney function. Emerging research indicates that 3PEHPC may enhance mitochondrial dynamics, which could be key in addressing some of the cellular dysfunctions characterizing SLE. By potentially optimizing cellular energy metabolism, 3PEHPC could play a vital role in improving overall health outcomes for patients suffering from this complex autoimmune disorder [111,112]. While current research is still in preclinical phases, initial findings from studies utilizing SLE models present promising improvements in renal function and reductions in autoantibody levels. Such encouraging results underscore the necessity for further clinical exploration to evaluate the efficacy and safety of 3PEHPC in human subjects, heralding the promise of novel therapeutic options for managing SLE in the near future [113,114].

### 6.7. Pioglitazone

Pioglitazone, a thiazolidinedione agent primarily used in the treatment of diabetes mellitus, exhibits intriguing properties that extend beyond glucose regulation. By binding to the mitochondrial electron transport chain complex 1, pioglitazone induces depletion of adenosine triphosphate (ATP) within cells, which may contribute to its therapeutic effects [115,116]. Notably, in models of systemic lupus erythematosus (SLE), pioglitazone has demonstrated a significant ability to inhibit autoimmune responses and improve nephritis. Research further reveals that it effectively restrains the proliferation of T-helper cells while considerably promoting the activity and increasing the number of T-regulatory cells [117]. As a PPARγ agonist, pioglitazone is being actively investigated for its potential to modulate immune responses and mitigate inflammation associated with lupus. By activating PPARγ, it may help restore balance within the immune system, potentially addressing the inflammatory pathways implicated in SLE. Initial clinical studies suggest that pioglitazone leads to improvements in nephritogenic activity and enhancements in T-regulatory cell functions, laying the groundwork for ongoing trials aimed at fully elucidating its therapeutic potential [118,119]. These investigations seek to establish the efficacy and safety profile of pioglitazone in patients with lupus, signaling the potential for a novel therapeutic option in managing this multifaceted autoimmune disorder and enhancing patient outcomes [120].

### 6.8. Piceid

Piceid, a bioactive compound derived from Reynoutria japonica, has garnered significant interest in the context of systemic lupus erythematosus (SLE) due to its promising therapeutic properties. Research indicates that Piceid effectively suppresses the formation of neutrophil extracellular traps (NETs), which are implicated in the pathogenesis of SLE and associated with heightened inflammatory responses [56,121]. In pristane-administered MRL–lpr murine models of SLE, Piceid not only alleviated symptoms but also demonstrated its capacity to inhibit reactive-oxygen-species-mediated NETosis, thereby diminishing the inflammatory processes that contribute to disease severity. While most of the current data derive from animal studies, which highlight its potential to mitigate SLE-related inflammation, translational research is actively underway to assess the efficacy of Piceid in human patients [122,123]. This research aims to bridge the gap between preclinical findings and clinical application, potentially paving the way for innovative therapeutic strategies to manage lupus effectively. By addressing the underlying mechanisms of inflammation, Piceid may represent a novel approach to improving outcomes for individuals affected by this complex autoimmune disorder [5,113].

### 6.9. Nestin

Nestin is an essential cytoskeletal protein that plays a significant role in kidney health, particularly in the context of podocytes, which are crucial for maintaining glomerular function. Constantly released in podocytes, nestin is closely associated with processes of podocyte damage, making it a vital biomarker in kidney pathology [124,125]. Importantly, it has the ability to modulate the release of nephrons by regulating mitochondrial autophagy and oxidative stress, thus providing a protective shield for podocytes against potential injury in systemic lupus erythematosus (SLE) nephritis. Current investigations into the clinical application of nestin focus on its role in enhancing mitochondrial health and cellular resilience within the nephric environment [126]. As researchers explore how nestin can mitigate mitochondrial dysfunction, its potential contributions to safeguarding renal function in SLE patients become increasingly evident. Ongoing experimental studies aim to delineate the protective effects of nestin on kidney cells comprehensively, with the hope of translating these findings into practical therapeutic strategies that could improve patient outcomes and preserve overall kidney health in individuals suffering from this complex autoimmune disease [127].

## 7. Conclusions

The exploration of mitochondrial-targeted treatments offers a promising direction for enhancing therapeutic options for systemic lupus erythematosus (SLE). Current treatment regimens often involve broad-spectrum immunosuppressive therapies, which can lead to serious side effects, including organ failure and increased susceptibility to infections. By focusing on mitochondrial function, there is potential not only to alleviate symptoms but also to address the underlying mechanisms of the disease.

Mitochondrial-targeted therapies, such as sirolimus, N-acetylcysteine, and Coenzyme Q10, demonstrate diverse mechanisms of action that could benefit different subsets of SLE patients. For instance, patients with renal involvement may particularly benefit from therapies that enhance mitochondrial function and reduce oxidative stress, while those experiencing more systemic manifestations might respond better to treatments that modulate immune cell activity.

To improve standard care and personalize therapy, we need a deeper understanding of mitochondrial dysfunction in SLE. This includes identifying specific mitochondrial biomarkers that could guide therapy selection based on individual patient profiles. Research should aim to determine which subsets of patients are most likely to benefit from specific mt-targeted agents and to clarify the optimal timing and combinations of these therapies with existing treatments.

Furthermore, future studies should focus on the long-term effects of these treatments, as well as their influence on quality of life and disease progression. By bridging our current knowledge gaps, we can aspire to personalize therapy for SLE patients through targeted mitochondrial interventions, ultimately improving clinical outcomes and patient satisfaction.

In conclusion, the insights provided in this review accentuate the critical role of mitochondria in driving immune dysregulation and inflammation in SLE, presenting mitochondria as a promising therapeutic target in the management of this complex autoimmune disorder. Continued research efforts and the development of innovative mitochondrial-focused therapies hold the potential to revolutionize treatment strategies for SLE and pave the way for personalized precision medicine approaches tailored to mitigate mitochondrial dysfunction and improve outcomes for patients with SLE.

## Figures and Tables

**Table 1:** Summary of mitochondrial function impairment in systemic lupus erythematosus.

Main Process	Mechanism/Process	Implications	Therapeutic Strategies
Reduction–Oxidation Alterations	Elevated mitochondrial reactive oxygen species (ROS) production	Impairment leads to cellular injury and release of mtDNA; perpetuates oxidative stress (OS)	Anti-oxidant therapies (e.g., NAC), targeting ROS production
Genetic Factors	Mitochondrial single nucleotide polymorphisms (SNPs)	Genetic susceptibilities to SLE; accumulation of autoantibodies and immune complexes (ICs)	Gene-targeted therapies; modulating mitochondrial functions
Mitochondrial Dysfunction in Immune Cells	Impaired mitophagy and increased mtROS in T-lymphocytes	Alters T-cell-signaling pathways; leads to T-lymphocyte exhaustion and autoantigen expression	mTORC1 inhibitors (e.g., rapamycin), Metformin
Innate Immune Response	Increased interferon alpha from activated immune complexes	Drives autoreactivity and inflammation; promotes autoantibody generation	Targeting interferon pathways; restoring mitophagy
NETosis and Inflammation	Mitochondrial DNA (mtDNA) release linked to NET formation	Contributes to inflammation via immune complex formation and exacerbates disease pathology	Scavenging agents for mtROS; controlling NET formation
Inflammasome Activation	Cryopyrin inflammasome linked to ROS and mtDNA release	Activates proinflammatory cytokines; exacerbates SLE progression	Targeting inflammasome pathways; enhancing mitophagy
B-Lymphocyte Dysfunction	mTORC1 activation linked to excessive autoantibody production	Drives differentiation of B-cells into plasmablasts, furthering autoimmune response	Inhibiting mTORC1; Metformin to regulate B-cell activity
Calcium (Ca^2+^) Dysregulation	Altered calcium efflux in T-lymphocytes	Disrupts normal immune signaling and function, contributing to T-cell dysregulation	Targeting pathways that regulate Ca^2+^ homeostasis

**Table 2:** Immunopathological impacts of mitochondrial dysfunction in SLE-immune cells.

Immune Cell Type	Mitochondrial Dysfunction Mechanism	Impact on Immune Function	Implications for SLE Pathogenesis
T-lymphocytes	Increased ROS production, impaired mitophagy	T-cell exhaustion, skewed immune response	Increased autoreactivity, disease exacerbation
B-lymphocytes	mTORC1 activation linked to excessive autoantibody production	Enhanced differentiation into plasmablasts	Amplified humoral response
Neutrophils	Impaired NETosis, excessive ROS generation	Dysfunctional clearance of pathogens, persistent inflammation	Sustained inflammatory response, tissue damage
Plasmacytoid dendritic cells	Increased interferon-alpha production linked to mitochondrial stress	Heightened type I interferon response and autoimmunity	Promotion of inflammation, anti-dsDNA autoantibody production

**Table 3:** Summary of mitochondrial-targeted therapies for systemic lupus erythematosus.

Therapy	Mechanism of Action	Demonstrated Effects
Sirolimus (Rapamycin)	m-TOR suppression, reducing T-lymphocyte function	Improved mitochondrial function; decreased protein levels in urine; enhanced renal function; reduced anti-ds-DNA antibody titers
N-acetylcysteine (NAC)	GSH precursor; anti-oxidant and ROS removal; m-TORC-1 suppression	Reduced anti-double stranded DNA antibody levels; alleviated SLE nephritis symptoms; decreased lupus activity
Coenzyme Q10 (CoQ10)	Anti-oxidant; neutralizes free radicals; facilitates electron transport	Decreased mitochondrial ROS production; reduced NETs activation and interferon alpha generation; improved immune response
Metformin	Normalizes redox balance; AMPK–m-TOR–STAT-3 pathway modulation	Decreased ROS generation; reduced mitochondrial DNA leakage; ameliorated SLE conditions; lowered corticosteroid dependence
Hydroxychloroquine (HCQ)	Targets mitochondrial anti-oxidant systems; immunomodulator	Decreased proliferation of T-helper cells; inhibited proinflammatory cytokines; potentially improved SLE symptoms
3PEHPC	Suppresses Rab-protein geranylgeranyltransferase	Improved mitochondrial dynamics; decreased anti-nuclear antibody production; alleviated nephritis symptoms
Pioglitazone	Binds to mitochondrial electron transport chain complex	Inhibited autoimmune response; improved nephritis symptoms; promoted T-reg activity
Piceid	Suppresses ROS-mediated NET formation	Alleviated SLE symptoms in murine models
Nestin	Modulates mitochondrial autophagy and oxidative stress	Protects podocytes from injury; potentially alleviates symptoms associated with SLE nephritis
